# A Latent Class Analysis of Hallucination-like Experiences in Nonclinical Adults from Jammu and Kashmir

**DOI:** 10.1093/schizbullopen/sgag038

**Published:** 2026-09-16

**Authors:** Amir Sultan, Arsheena Manzoor, Ulfat Jaan

**Affiliations:** Department of Psychology, University of Kashmir, Hazratbal, Srinagar, J&K, 190006, India; Department of Psychology, University of Kashmir, Hazratbal, Srinagar, J&K, 190006, India; Jamia Millia Islamia; Head Department of Psychology, Government Degree College, Duderhama, J&K, 191201, India

**Keywords:** hallucinations, psychosis, latent class analysis, quasi-continuum model, Jammu and Kashmir

## Abstract

**Background and Hypothesis:**

Hallucination-like experiences (HLE) are increasingly conceptualized as existing on a continuum ranging from normative perceptual anomalies to clinically significant psychotic symptoms. Although substantial research has examined HLE in nonclinical populations, comparatively limited evidence is available from South Asian contexts, particularly using person-centered approaches. The present study investigated the latent structures of HLE in a nonclinical sample from Jammu and Kashmir using the Launay–Slade Hallucination Scale, extended version (LSHS-E). It was hypothesized that distinct latent subgroups differing primarily in the severity of HLE endorsement would emerge within the sample.

**Study Design:**

A cross-sectional design was employed with 382 adults recruited through online and paper–pencil survey administration. Confirmatory factor analysis supported the four-factor structure of the LSHS-E, demonstrating acceptable model fit (Comparative Fit Index (CFI) = 0.929, Tucker Lewis Index (TLI) = 0.913, Root Mean Square Error of Approximation (RMSEA) = 0.068, Standardized Root Mean Square Residual (SRMR) = 0.044). The scale demonstrated excellent overall internal consistency (α = .90), with subscale reliabilities ranging from acceptable to good (α = .73-.79). Item-level analyses indicated generally low endorsement of HLE within the sample, although intrusive thoughts and vivid imagery-related experiences were comparatively more common.

**Study Results:**

Latent class analysis identified a three-class solution comprising low endorsement (47.4 %), moderate endorsement (31.0 %), and high endorsement (21.6 %) profiles. The classes differed primarily in severity rather than qualitatively distinct symptom patterns, supporting quasi-continuum models of psychosis. Demographic variables did not significantly predict latent class membership.

**Conclusions:**

Overall, the findings support the quasi-continuum and person-centered conceptualization of hallucination-proneness and contribute culturally informed evidence regarding the heterogeneity within a South Asian nonclinical population.

## Introduction

Hallucination-like experiences (HLE) are perceptual experiences occurring in the absence of corresponding external stimuli and include phenomena such as intrusive thoughts, vivid mental imagery, auditory experiences, visual anomalies, sensed presence experiences, and multisensory perceptions.^1^^,^^2^ Traditionally associated with schizophrenia–spectrum disorders, evidence indicates that HLE also occur in the general population.^3^ Contemporary psychosis research conceptualizes HLE as existing along a continuum ranging from mild to severe psychotic symptoms.^4^^,^^5^ This dimensional perspective emphasizes variability in intensity, frequency, distress, and functional impact rather than strict categorical distinctions between “normality” and “psychopathology”.^1^^,^^6^

Research in the nonclinical population has become important because it enables the study of psychosis-like experiences (PLE) without the confounding effect of chronic illness, hospitalization, medication, or severe impairment.^3^^,^^7^ Epidemiological studies demonstrate that HLE are relatively common and are often transient, insight-retained, and minimally distressing.^8^^,^^9^ Large population based studies have similarly demonstrated substantial variation in frequency and phenomenology of hallucinatory experiences among individuals without a diagnosed psychotic disorder.^10^ Collectively, these findings suggest that the clinical significance of HLE depends less on their mere presence and more on associated distress, appraisal, and co-occurring psychopathology.

Theoretical explanations of HLE draw upon cognitive and predictive processing frameworks. Predictive processing theories propose that hallucinations may arise when internally generated representations become insufficiently constrained by external sensory input, leading to the external attribution of internally generated experiences.^7^ Related cognitive accounts emphasize deficits in source monitoring, self-monitoring, and reality discrimination among hallucination-prone individuals.^1^^,^^11^ Supporting these models, studies indicated elevated HLE are associated with reality-monitoring errors and mental imagery vividness across sensory modalities.^7^^,^^12^ Phenomenological perspectives similarly suggest that many nonclinical hallucinations resemble internally generated imagery rather than fully externalized psychotic hallucinations.^13^^,^^14^

Research further indicates that HLE are multidimensional rather than unitary constructs.^2^^,^^15^ Factor analytic studies using the Launay–Slade Hallucination Scale and its extended versions (LSHS-E) have identified dimensions such as intrusive thoughts, vivid daydreams, multisensory experiences, sleep-related hallucinations, and auditory or visual hallucinations.^16^^,^^17^ These dimensions differ in psychological significance, with intrusive thoughts and multisensory experiences showing stronger associations with distress and psychosis-proneness than vivid daydreaming.^2^ Such findings support the view that hallucination-proneness reflects heterogeneous experiences associated with varying degrees of psychological vulnerability.^5^

Recognition of heterogeneity has contributed to the increasing use of person-centered approaches such as latent class analysis (LCA). Person-centered refers to approaches that identify unobserved subgroups of individuals based on their patterns of responses across multiple indicators, in contrast to variable-centered approaches that examine relationships among variables across the sample as a whole. Previous research using person-centered approaches has identified groups characterized by relatively low endorsement as well as groups showing broader or elevated patterns of PLE.^6^^,^^18^ Such findings complement dimensional models of psychosis by highlighting heterogeneity in the configuration of experiences across individuals.

Beyond their phenomenological and psychological characteristics, HLE may also vary in how they are interpreted and appraised within different sociocultural contexts. Although many HLE are transient and non-distressing, persistent or clinically significant experiences are more likely when accompanied by emotional dysregulation, trauma, distress, or co-occurring psychopathology.^19^^,^^20^ Importantly, the occurrence of an unusual perceptual experience and the meaning attributed to that experience are not necessarily equivalent. Research has shown that individuals may differ in their emotional responses and everyday interpretations.^5^ Religious and other belief systems may also provide frameworks through which unusual experiences are understood and assigned meaning. Consistent with this possibility, Hinterbuchinger et al. found that esoteric believers reported elevated PLE without corresponding increases in distress.^21^ Cross-national research further demonstrates significant variability in the prevalence and expression of HLE across sociocultural settings.^17^^,^^22^ These findings suggest that sociocultural context may be relevant not necessarily because it determines whether an HLE occurs, but because it may influence how such an experience is described, interpreted, communicated, and appraised.

This consideration is particularly relevant when examining HLE in culturally diverse and understudied populations. India represents a highly heterogeneous sociocultural context in which religious, linguistic, familial, and community environments vary considerably across regions. Consequently, findings derived predominantly from western or culturally homogenous samples may not fully capture the range of HLE endorsement and phenomenological configurations present in Indian populations. For example, Sahu and colleagues validated the LSHS among healthy Indian individuals, providing an important foundation for the assessment of hallucination proneness within the Indian context. Nevertheless, comparatively limited research has examined the heterogeneity and latent structure of HLE across specific Indian sociocultural settings.^23^

Jammu and Kashmir represents one such relatively understudied setting. The region provides a distinct sociocultural context in which religious, linguistic, familial, and community environments may shape the ways in which unusual psychological experiences are communicated and understood.^24^^,^^25^ Such contextual considerations are relevant to the study of HLE because religious and culturally shared explanatory frameworks can potentially influence the meaning attributed to unusual experiences.^21^ However, these contextual considerations should not be interpreted as evidence that cultural or religious factors cause HLE. Rather, they highlight the importance of examining HLE empirically within populations whose sociocultural contexts have been comparatively underrepresented in the literature.

Recent research has begun to address PLE within the Kashmiri population. For instance, a study of delusion-like experiences among nonclinical emerging adults demonstrated the relevance of person-centered approaches to understanding PLE within the local population.^26^ The latent structure of HLE, particularly across different phenomenological domains assessed by the LSHS-E, remains insufficiently characterized in nonclinical population from the region. Examining such patterns may therefore extend both the Indian literature on HLE and the broader cross-cultural literature on PLE.

Importantly, the present study does not directly assess religious beliefs, spiritual practices, supernatural explanations, cultural models, or subjective interpretations of HLE; therefore, the sociocultural characteristics of Jammu and Kashmir are treated as contextual rather than explanatory. The study instead aims to empirically characterize heterogeneity in HLE endorsement within a nonclinical sample using the LSHS-E, by identifying latent classes, examining differences between them, and assessing demographic predictors of class membership. These classes are not assumed to represent culturally meaningful categories but provide an empirical basis for future research on the role of sociocultural context and subjective appraisal.

## Methods

### Participants

We adopted a cross-sectional design comprising 452 adults aged 18 years and above, including both males and females, between February and April 2026. Data were collected using convenience sampling through a hybrid approach combining online and paper–pencil administration. The online survey link, hosted on Google Forms, was circulated through social media platforms, college and university networks, and the researchers’ professional contacts. Paper-based surveys were administered to individuals with limited internet access or a preference for physical completion.

To ensure a community-based, nonclinical sample, individuals reporting a formal diagnosis of a neurological, substance use, or psychiatric disorder were excluded. Information regarding these diagnoses was obtained through a self-report screening item included in the demographic section of the questionnaire. While this self-report approach could not be verified via secondary clinical interviews or medical record reviews due to logistical constraints, it is consistent with prior research evaluating the LSHS-E in similar populations.^22^^,^^23^ The presumptive nature of the study is noted as a limitation. In addition, participants from demographic categories with insufficient representation for meaningful statistical interpretation, as well as those residing outside Jammu and Kashmir, were excluded from the analyses. Consequently, 31 participants with a diagnosed neurological, substance use, or psychiatric disorder, 38 participants not domiciled in Jammu and Kashmir, and 1 participant belonging to an underrepresented religious category (Sikhism) were excluded, resulting in a final sample of 382 participants. The obtained sample exceeded the recommended minimum sample size for LCA.^27^

The mean age of the sample was 30.9 years (SD = 11.1). As shown in Table 1, 196 (51.3%) participants were females and 186 (48.7%) were males. Regarding educational qualification, 206 (53.9%) participants were undergraduates (UGs), while 176 (46.1%) were postgraduates (PGs). In terms of occupation, 215 (56.3%) participants were not working and 167 (43.7%) were working. Considering marital status, 255 (66.8%) participants were single and 127 (33.2%) were married. A total of 263 (68.8%) participants belonged to nuclear families, while 119 (31.2%) belonged to joint families (multi-generational households or related family units living together). Regarding domicile, 182 (47.6%) participants resided in rural areas, whereas 200 (52.4%) were from urban areas. Considering socioeconomic status (SES), 84 (22.0%) participants belonged to below poverty level (BPL) households, while 298 (78.0%) belonged to above poverty level (APL) households. With respect to religion, 324 (84.8%) participants identified as Muslims whereas 58 (15.2%) identified as Hindus.

**Table 1:** Demographic Characteristics of the Sample (*N* = 382)

Variable		*N* (%)
Gender	Male	186 (48.7)
	Female	196 (51.3)
Education	UG	206 (53.9)
	PG	176 (46.1)
Occupation	Working	167 (43.7)
	Not working	215 (56.3)
Marital status	Married	127 (33.2)
	Single	255 (66.8)
Family type	Nuclear	263 (68.8)
	Joint	119 (31.2)
Domicile	Rural	182 (47.6)
	Urban	200 (52.4)
Socioeconomic status	BPL	84 (22.0)
	APL	298 (78.0)
Religion	Islam	324 (84.8)
	Hindu	58 (15.2)

Abbreviations: UG = undergraduate; PG = postgraduate; BPL = below poverty line; APL = above poverty line.

### Measures

#### Hallucination-like Experiences

We used the Launay–Slade Hallucination Scale-Extended Version (LSHS-E) to assess HLE.^28^^,^^29^ The scale comprises 16 items spanning auditory, visual, tactile, and olfactory modalities, as well as hypnagogic and hypnopompic phenomena vivid daydreams, and the experience of sensed presence. Respondents rate each item on a five-point Likert scale ranging from 0 (*certainly does not apply to me*) to 4 (*certainly applies to me*), with higher aggregate scores reflecting a greater propensity for hallucinatory experiences. Previous studies have demonstrated adequate psychometric properties of the scale across clinical and nonclinical populations,^17^^,^^22^^,^^23^ and the scale has been extensively employed in population-based investigations of subclinical psychotic phenomena.

In the present sample, the reliability and validity of the LSHS-E were examined. Confirmatory factor analysis (CFA) was conducted to examine the four-factor structure proposed by Sahu and colleagues,^23^ comprising Intrusive Thoughts, Vivid Daydreams, Multisensory HLE, and Auditory–Visual HLE. Although the early principal component analysis of modified scale variants yielded alternative configurations,^28^ the retained 4-factor structure aligns with the formalized 16-item LSHS-E^29^ and has received extensive empirical support, including cross-cultural invariance across 10 countries and between clinical and nonclinical cohorts.^2^^,^^22^ Selecting this baseline factor structure also accounts for recognized cultural and linguistic variations in HLE expression^30^ while maintaining continuity with prior implementations in comparable regional samples.^23^

The correlated four-factor model demonstrated acceptable fit to the data, χ^2^ (98) = 273.00, *P* < .001, CFI = 0.929, TLI = 0.913, SRMR = 0.044 and RMSEA = 0.068, 90% CI [0.059, 0.078]. Standardized factor loadings ranged from 0.536 to 0.805, indicating moderate to strong item-factor relationships across all four latent constructs. All freely estimated factor loadings were statistically significant (*P* < .001); however, given the relatively large sample size, statistical significance alone provides limited evidence of factorial validity, and the magnitude of the standardized loadings is more informative in this regard. Inter-factor correlations were positive and statistically significant, ranging from 0.580 to 0.969. Particularly high correlations were observed between Multisensory HLE and Auditory–Visual HLE (r = .969), which is above conventional thresholds for discriminant validity. Rather than indicating an untenable measurement model, this overlap reflects the fact that auditory–visual experiences represent the primary constituent of multisensory phenomena, pointing to a strong underlying general hallucination-proneness factor. To formally test this, a hierarchical/second-order factor model was evaluated alongside competing specifications, following established precedents^17^; complete fit statistics and model comparisons are provided in the supplementary file.

Subsequently, Cronbach’s alpha^31^ coefficients indicated acceptable to good reliability: Intrusive Thoughts (α = .73), Vivid Daydreams (α = .79), Multisensory HLE (α = .78), and Auditory–Visual HLE (α = .79). The overall LSHS-E demonstrated excellent internal consistency (α = .90). Collectively, these findings support the factorial validity and internal consistency reliability of the LSHS-E within the present sample.

### Procedure

The questionnaire was administered through two parallel channels. To enhance data quality and minimize duplicate participation, the online form was configured to restrict responses to one submission per account. In addition, to minimize missing data, all items in the online form were configured as mandatory and paper–pencil-based questionnaires were checked for completeness at the time of data collection. No missing data were found.

Before participation, all respondents were provided with an informed consent form describing the purpose of the study, the voluntary nature of participation, confidentiality of response, the absence of financial remuneration, and the right to withdraw at any stage without penalty. No monetary compensation, course credit, academic incentive, or any other form of remuneration was provided to any participant in either administration format. Participation was contingent upon informed consent. The questionnaire required approximately 10 to 15 minutes to complete.

### Statistical Analyses

We computed descriptive statistics to characterize the sample, CFA to evaluate the validity of the theoretically specified four-factor structure, Cronbach’s α to assess reliability, and LCA on the 16 items of the LSHS-E using the Jamovi software.^32^ LCA is a person-centered statistical method used to identify mutually exclusive, exhaustive, and latent subgroups within a population based on a set of indicators.^27^ Thus, whereas CFA evaluates the four-factor measurement structure at the variable level, LCA addresses whether individuals form discrete subgroups characterized by different patterns of item endorsement. The two analyses are therefore complementary, with the CFA establishing the validity of the item set prior to its use in identifying latent subgroups. Analyses were conducted using the *snowLatent* module in Jamovi. The estimation routines were based on the *glca* R package, which implements latent class modeling procedures.^33^^,^^34^

Models were estimated beginning with a two-class solution and progressing to increasingly complex models. Model selection was guided by the log-likelihood, likelihood ratio statistics (*G*^2^), Akaike Information Criterion (AIC), Consistent AIC (CAIC), Bayesian Information Criterion (BIC), entropy, interpretability of the latent classes, and adequacy of class proportions.

Finally, we performed multinomial logistic regression analyses to examine whether demographic variables predicted latent class membershipA significance threshold of α = .05 was adopted throughout the analyses. Full details of the CFA, LCA model-fit statistics, and item probability profiles across all estimated solutions are given in supplementary files.

## Results

### Mean, SD, and Skewness–Kurtosis of Scores on LSHS-E

The mean total score of the LSHS-E was 21.0 (*SD* = 13.7). Examination of the distributional characteristics of the total score including skewness (z = 3.14) and kurtosis (z = -2.63), suggested modest deviations from normality. However, these deviations are considered acceptable in large samples and are characteristic of psychological constructs assessed in community populations.^35^ Crucially, because LCA was estimated directly from item responses rather than continuous sum scores, the model does not assume multivariate normality across the sample, but rather local independence of item indicators given latent class membership. Such distributional deviations do not violate the assumptions of LCA, which requires conditional normality within classes rather than global normality across the full sample.

A heatmap was constructed (Figure 1) to illustrate the percentage distribution of responses across the 16 items of the LSHS-E. Responses were predominantly concentrated in the *certainly does not apply* category across most items, indicating generally low endorsement of HLE within the sample. The floor-effect pattern was particularly pronounced for items assessing rare sensory anomalies, specifically Q10 (61%), Q16 (55.8%), Q15 (55.5%), and Q9 (52.9%) which exhibited the greatest item-level positive skew. Rather than obscuring model estimation, this low base-rate endorsement is a characteristic feature of subclinical screening in the general population; it provided clear anchor points for distinguishing the nonclinical baseline, thereby facilitating clean class separation and contributing to the high-level variability, with higher proportions of participants endorsing the *possibly applies* and *certainly applies* categories.

**Figure 1:**
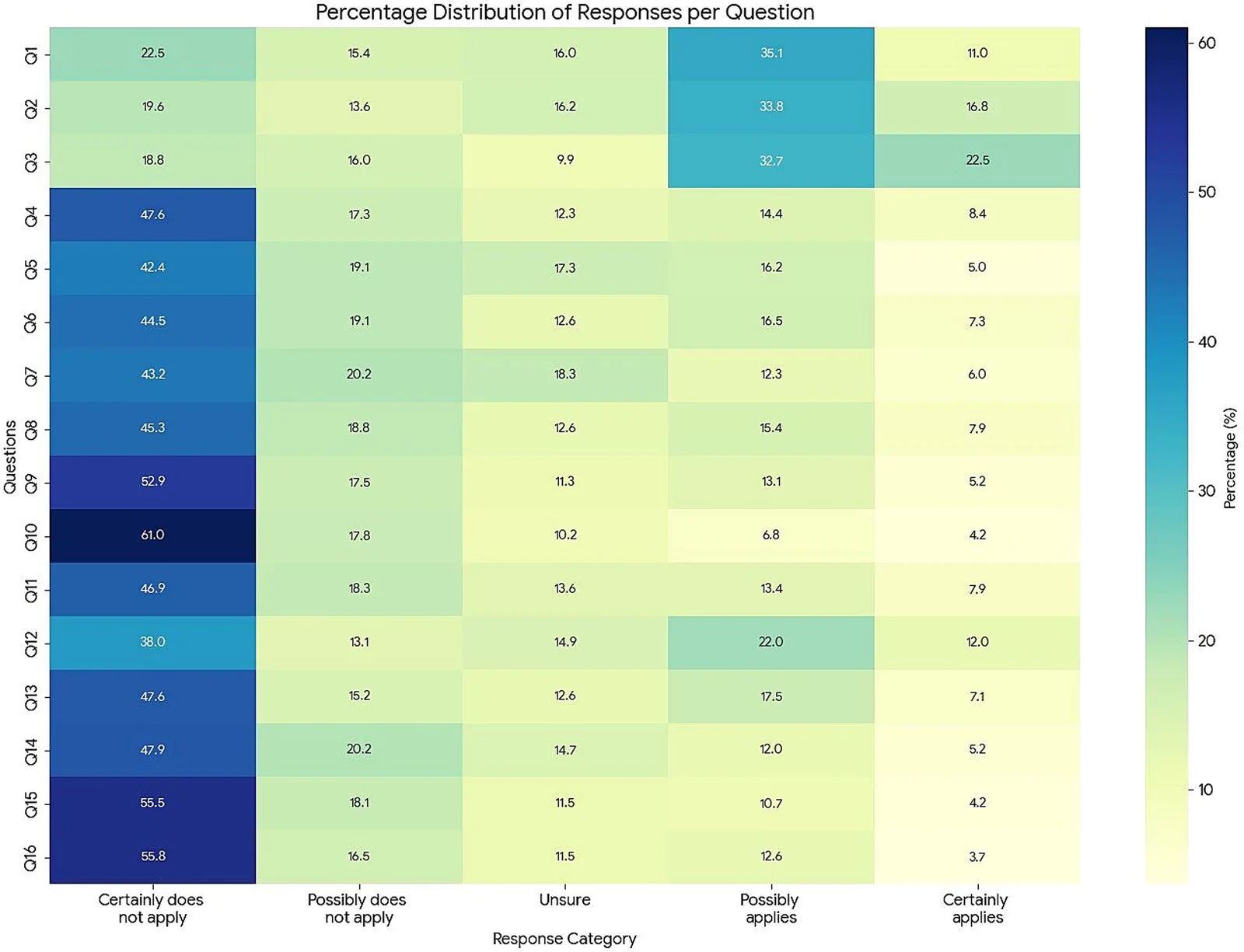
Percentage Distribution of Responses across the 16 Items of the Launay–Slade Hallucination Scale-Extended (LSHS-E).

### Latent Class Analysis

LCA was conducted using participant responses on the 16 LSHS-E items. As shown in Table 2, comparison of the 2-class, 3-class, and 4-class solutions indicated that the 3-class model provided the most interpretable and parsimonious representation of the data, evidenced by lower BIC (15932) and CAIC (16066) values and higher entropy (0.952). Although the 4-class model demonstrated a lower AIC value, simulation work on class-enumeration in mixture models demonstrates that AIC is systematically biased toward over-extraction by selecting extra classes relative to the true generating model more often than BIC.^27^Therefore, preferring the lower BIC and CAIC and higher entropy of the three-class solution over the marginally lower AIC of the higher-class specification is consistent with best-practice guidance in this literature. The likelihood ratio difference test also supported the 3-class model, ΔG^2^ = 512, *P* < .001. The three-class model (G^2^ = 10 341, df = 187) also yielded optimal classification precision alongside a straightforward, theoretically coherent ordering of low, moderate, and high HLE endorsement. Therefore, the 3-class solution was retained for further interpretation.

**Table 2:** Fit Indices for Latent Class Models

Model	Log-likelihood	df	G^2^	AIC	CAIC	BIC	Entropy	Class membership
2-Class	−7610	252	10 842	15 478	16 116	15 978	0.938	47.5/52.5
3-Class	−7359	187	10 341	15 107	16 066	15 872	0.952	21.6/31/47.4
4-Class	−7178	98	13 092	14 874	16 155	15 896	0.940	26.5/34.8/23.3/15.5

Abbreviations: AIC = Akaike Information Criterion; BIC = Bayesian Information Criterion; G^2^ = likelihood ratio statistic.

### Latent Class Profile Plot

As shown in Figure 2, the final three classes represented distinct response profiles derived from the 16 items and differed primarily in the severity of HLE endorsement. Class 1 (high endorsement) comprised 21.6% of the sample and demonstrated consistently elevated expected scores across nearly all items, indicating high levels of HLE. This class showed particularly strong endorsement of items Q1-Q3, Q6, Q11, and Q12, suggesting greater intensity of intrusive thoughts, vivid perceptual experiences, and Multisensory HLE.

**Figure 2:**
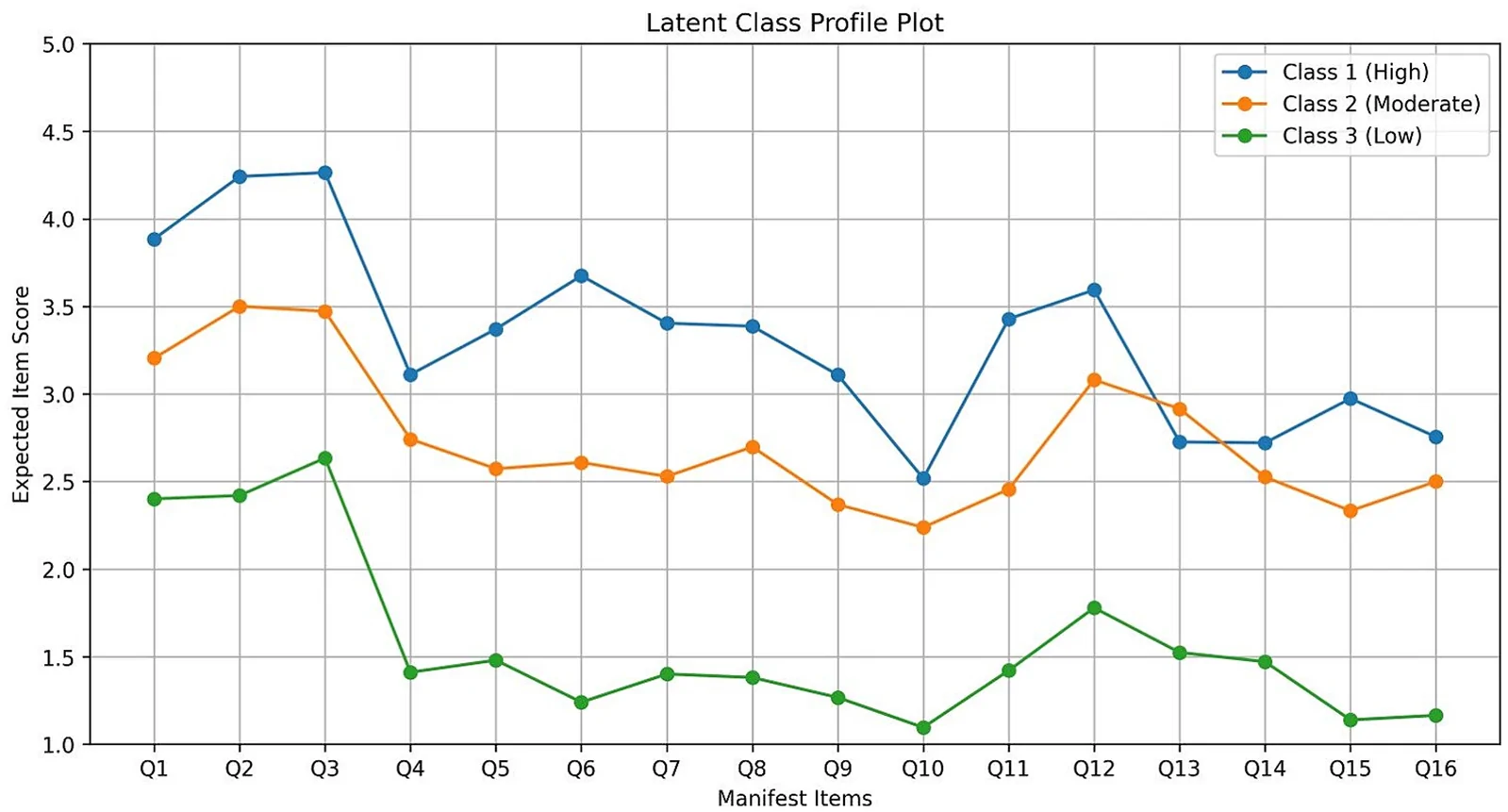
Latent Class Profile Plot of the Given Population.

Class 2 (moderate endorsement), representing 31% of the sample, exhibited intermediate expected scores across the manifest items. Although the general response pattern resembled that of the high endorsement class, the magnitude of endorsement was comparatively lower, reflecting moderate levels of HLE.

Class 3 (low endorsement), the largest subgroup comprising 47.4% of the sample, showed consistently low expected scores across all indicators, indicating minimal endorsement of HLE. This class demonstrated the lowest levels of auditory, visual, and Multisensory HLE as well as intrusive thought-related experiences. Overall, the latent profiles appeared to differ quantitatively rather than qualitatively, as all three classes displayed broadly similar response patterns with varying levels of endorsement.

### Demographic Predictors of Latent Class Membership

Multinomial logistic regression analyses were conducted to examine whether demographic variables predicted latent class membership, with Class 3 (low endorsement) used as the reference category. Results as shown in Table 3 indicate that none of the demographic variables significantly predicted membership in either Class 1 (high endorsement) or Class 2 (moderate endorsement) relative to the Class 3 (low endorsement class) (all *P >* .05). Specifically, for Class 1 versus Class 2, odds ratios ranged from 0.54 to 2.31, suggesting that variations in gender, education, occupation, marital status, family type, domicile, SES, and religion were not reliably associated with increased or decreased likelihood of belonging to the high endorsement class.

**Table 3:** Multinomial Logistic Regression Predicting Latent Class Membership

Predictor	Comparison	*B*	SE	OR	*P*
Gender	Class 1 vs. Class 3	0.027	0.623	1.028	.965
Education	Class 1 vs. Class 3	−0.041	0.488	0.960	.934
Occupation	Class 1 vs. Class 3	0.562	0.587	1.755	.339
Marital status	Class 1 vs. Class 3	0.835	0.809	2.305	.304
Family type	Class 1 vs. Class 3	−0.314	0.687	0.731	.649
Domicile	Class 1 vs. Class 3	0.014	0.500	1.014	.978
SES	Class 1 vs. Class 3	−0.620	0.647	0.538	.339
Religion	Class 1 vs. Class 3	0.012	0.743	1.012	.988
Gender	Class 2 vs. Class 3	−0.499	0.347	0.607	.152
Education	Class 2 vs. Class 3	−0.152	0.393	0.859	.700
Occupation	Class 2 vs. Class 3	0.768	0.438	2.155	.082
Marital status	Class 2 vs. Class 3	0.221	0.425	1.248	.603
Family type	Class 2 vs. Class 3	0.030	0.396	1.030	.940
Domicile	Class 2 vs. Class 3	−0.058	0.410	0.943	.887
SES	Class 2 vs. Class 3	−0.537	0.446	0.584	.230
Religion	Class 2 vs. Class 3	0.098	0.514	1.103	.849

Abbreviations: Reference Category = Class 3 (Low Endorsement); B = unstandardized logistic regression coefficient; SE = standard error; OR = odds ratio; SES = socioeconomic Status.

Similarly, comparisons between Class 2 and Class 3 revealed no statistically significant predictors, although occupation demonstrated a relatively stronger positive association (OR = 2.16, *P* = .082), indicating that certain occupational groups may have shown a greater tendency toward moderate endorsement of HLE; however, this effect did not reach statistical significance. Overall, these findings suggest that the observed latent classes were not meaningfully differentiated by demographic characteristics, implying that variation in HLE within the sample was relatively independent of socio-demographic background of this sample.

In summary, the LCA identified three distinct latent classes characterized by low, moderate, and high endorsement of HLE. The three-class solution demonstrated good statistical fit, high entropy, meaningful interpretability, and clear differentiation among classes, with profiles differing primarily quantitatively rather than qualitatively, while the demographic variables did not significantly predict latent class membership.

## Discussion

### Heatmap of Responses on the LSHS-E

The present study examined latent subgroups of HLE within a nonclinical sample using LCA based on responses to the 16 items of the LSHS-E. The heatmap of item responses (Figure 1) revealed a hierarchical pattern of endorsement consistent with quasi-continuum models of psychosis.^36^ Overall, responses were largely concentrated in the “certainly does not apply” category, indicating generally low endorsement of HLE within the sample. This pattern was particularly evident for items reflecting sensory anomalies and is consistent with previous findings suggesting that severe hallucinatory experiences remain comparatively infrequent in the general population.^1^^,^^6^

In contrast, items Q1-Q3 demonstrated comparatively greater variability in endorsement, with higher proportions of participants selecting “possibly applies” or “certainly applies”. These items primarily reflect lower-threshold experiences such as vivid imagery, intrusive thoughts, and imaginative involvement, which have been described as relatively common within clinical and nonclinical populations and may represent normative cognitive-perceptual processes rather than direct indicators of psychopathology.^36^

Notably, even the high endorsement subgroup demonstrated relatively lower endorsement of voice-hearing items compared to intrusive thought and multisensory experiences. This finding is consistent with evidence suggesting that nonclinical hallucination-proneness may reflect elevated cognitive-perceptual sensitivity rather than psychotic disorder itself.^2^^,^^16^ Consistent with this interpretation, hallucinations in nonclinical populations can frequently occur across multiple sensory modalities and vary substantially in distress, insight, and interpretation.^10^

### Latent Subgroups of HLE

The findings supported a three-class solution comprising low endorsement, moderate endorsement, and high endorsement profiles. Importantly, these classes differed primarily in the severity of endorsement rather than qualitatively distinct symptom structures. Although minor deviations from this overall severity gradient were observed for items 12 and 13, these were insufficient to support a qualitatively distinct interpretation of latent classes. Nevertheless, replication in independent samples could help determine whether these item-specific deviations represent stable features of the latent profiles or sample-specific variation.

The low endorsement subgroup demonstrated minimal endorsement across intrusive thought, auditory, visual, and multisensory items, suggesting relatively normative perceptual functioning. This finding aligns with epidemiological studies indicating that although mild anomalous experiences are relatively common, persistent, or severe hallucinatory experiences remain uncommon in nonclinical populations.^1^^,^^6^^,^^19^ Similarly, García-Ptacek et al. reported that perceptual anomalies in healthy individuals are often transient, insight-retained, and non-distressing.^8^

The moderate endorsement subgroup, comprising approximately one-third of the sample, demonstrated intermediate levels of endorsement across modalities. This profile resembles the “multisensory HLE” subgroup identified by Preti et al. and supports the view that hallucination-proneness exists along a continuum rather than within discrete pathological categories.^6^ Previous research further suggests that multisensory anomalous experiences may occur in psychologically healthy individuals without significant impairment or distress.^37^ Likewise, Waters et al. proposed that certain sensory anomalies may reflect normal variations in consciousness and REM-intrusion processes rather than psychosis itself.^9^

The high endorsement subgroup demonstrated consistently elevated endorsement across most items, particularly intrusive thoughts and multisensory experiences. Although this profile resembles previously identified high hallucination-proneness groups,^6^ existing literature suggests that such individuals may reflect increased cognitive-perceptual sensitivity and schizotypal vulnerability rather than a categorical psychotic disorder.^2^^,^^10^^,^^16^

The elevated endorsement of intrusive thought and multisensory items within this subgroup is also consistent with cognitive models of hallucinations emphasizing source-monitoring deficits and predictive processing disturbances. These models propose that internally generated cognitive events may be misattributed to external sources, contributing to hallucinatory experiences.^1^^,^^7^ Additional support comes from Smailes et al., who found associations between visual HLE, pareidolia-proneness, and reality discrimination errors.^12^

The identification of these latent subgroups supports previous evidence that HLE are distributed across nonclinical populations rather than being restricted to psychiatric disorders.^1^^,^^36^ Methodologically, the findings further demonstrate the utility of LCA in identifying heterogeneous profiles of hallucination-proneness, supporting contemporary person-centered approaches to psychotic-like experiences.^4^

### Demographic Variables and HLE

Another important finding was that demographic variables did not significantly predict latent class membership. Gender, education, occupation, marital status, family type, domicile, SES, and religion failed to reliably differentiate individuals across the identified profiles. These findings diverge somewhat from Scott et al., who reported modest demographic associations with psychotic-like experiences,^38^ but are more consistent with studies suggesting that HLE are broadly distributed across populations and are not strongly determined by demographic variables alone.^2^^,^^6^ All participants had at least a UG-level education, and the sample was predominantly Muslim (84.8%), resulting in limited representation across educational and religious categories. This restricted variation may have reduced the ability to detect demographic differences in latent class membership that could emerge in more heterogeneous and representative samples. Moreover, similar findings have also been reported in related research on delusion-proneness within comparable samples from the same region.^26^ Instead, previous literature increasingly highlights cognitive, emotional, and trauma-related factors as potentially important correlates of hallucination-proneness beyond socio-demographic background.^3^^,^^39^

### Dimensional Versus Categorical Models of Psychosis

A particularly important finding of the present study was that the latent classes differed primarily in severity rather than in qualitatively distinct symptom patterns. Although LCA identified distinct endorsement profiles, the overall response patterns across classes remained broadly similar, suggesting quantitative rather than categorical differences in hallucination-proneness. These findings are therefore more consistent with quasi-continuum conceptualizations of psychosis than with strictly categorical models.^1^^,^^36^ The findings are also consistent with Rogers et al., who proposed that hallucination-proneness reflects graded differences in internally generated imagery processes across sensory modalities.^7^ In line with previous literature, the present results further indicate that psychologically healthy individuals may exhibit varying degrees of hallucination-proneness without meeting criteria for a psychotic disorder.^2^^,^^8^^,^^10^^,^^20^ Accordingly, the identification of a high endorsement subgroup within a nonclinical sample supports the notion of an “extended psychosis phenotype”, wherein unusual perceptual experiences may reflect varying degrees of vulnerability rather than discrete pathological categories.^21^

### South Asian Cultural Context

The findings should also be interpreted within the broader South Asian sociocultural context. Although Siddi et al. demonstrated that hallucination-proneness represents a multidimensional and cross-culturally stable construct^22^; they also reported important cross-national differences in the prevalence and expression of HLE, suggesting a significant role of sociocultural influences. Within many South Asian settings, unusual perceptual experiences may be interpreted through spiritual, religious, or supernatural explanatory frameworks rather than exclusively biomedical perspectives. Bhavsar and Bhugra further argued that religious and culturally shared belief systems can shape the meaning and interpretation of psychotic-like experiences, thereby influencing whether such experiences are perceived as pathological or culturally acceptable.^40^

## Limitations

Several limitations should be considered. First, reliance on self-report measures may have introduced response bias and variation in the interpretation of HLE items.^14^ In addition, exclusion of participants with psychiatric, neurological, or substance-use disorders was based on a single self-report item rather than structured clinical assessment. Thus, the sample should be regarded as nonclinical by self-report rather than clinically verified.

Second, the sample was predominantly Muslim (84.8%) and relatively highly educated, with all participants having at least UG-level education. This restricted demographic variation may limit generalizability and reduce the ability to detect demographic differences in latent class membership. The cultural and religious context discussed here should therefore be regarded as contextual rather than explanatory, as these factors were not directly assessed.

Third, although the four-factor LSHS-E model showed acceptable fit, the very high correlation between the Multisensory HLE and Auditory–Visual HLE factors (r = .969) indicates substantial overlap and raises concerns regarding discriminant validity. Further evaluation of this structure in independent samples is warranted.

Finally, the cross-sectional design precludes conclusions about the temporal stability or developmental course of the latent classes. Longitudinal research incorporating measures of trauma, dissociation, emotional appraisal, reality monitoring, and psychological distress could clarify factors associated with class membership and determine whether greater HLE endorsement persists over time.^19^^,^^20^

## Conclusion

Overall, the present study supports quasi-continuum and person-centered conceptualizations of HLE within nonclinical populations. The identification of low, moderate, and high endorsement profiles suggests that HLE vary primarily in severity rather than in qualitatively distinct symptom structures, reinforcing continuum-based models of psychosis. The findings further highlight the importance of cognitive, phenomenological, and sociocultural factors in understanding hallucination-proneness outside clinical settings.

## Declarations

Ethical Considerations: The study was conducted in compliance with the Declaration of Helsinki^41^ and the ethical standards of the American Psychological Association.^42^ Institutional ethical approval was obtained from Government Degree College, Ganderbal, Kashmir, prior to data collection. All responses were anonymized prior to the analysis, and identifying information was not retained beyond the minimum necessary for the study. Given the non-invasive, self-report nature of the protocol and the absence of foreseeable participant risk, the study was classified as presenting minimal ethical concerns.

As the questionnaire assessed unusual perceptual and psychological experiences, participants were also provided with mental health support information at the end of the survey. Specifically, respondents who experienced discomfort or distress following participation were advised to contact Tele-MANAS mental health helpline services for psychological support and assistance.

## Supplementary Material

Supplementary_Files_HLE_sgag038

## Data Availability

The data can be made available by the corresponding author upon reasonable request.
