# Supplementary material for: A Latent Class Analysis of Hallucination-like Experiences in Nonclinical Adults from Jammu and Kashmir
Source: Schizophr Bull Open. 2026 Sep 16;7(1):sgag038. doi: 10.1093/schizbullopen/sgag038 (PMC13653665; doi:10.1093/schizbullopen/sgag038)
Supplement: Supplementary file 1 [file supplementary_files_hle_sgag038.docx]

**Supplementary Files**

**Title: A Latent Class Analysis of Hallucination-Like Experiences in Non-Clinical Adults from Jammu and Kashmir.**

**Corresponding Author:** Amir Sultan, PhD, Department of Psychology, University of Kashmir, Hazratbal, Srinagar, J&K, India. Email: [ibnmuhammadsultan@gmail.com](mailto:ibnmuhammadsultan@gmail.com) ORCID ID: 0000-0002-6614-5582.

Arsheena Manzoor, Doctoral Candidate, Department of Psychology, University of Kashmir, Hazratbal, Srinagar, J&K, India. Email: [arsheenamanzoor@gmail.com](mailto:arsheenamanzoor@gmail.com) ORCID ID: 0009-0001-3557-3314.

Dr. Ulfat Jaan, HOD Department of Psychology, Government Degree College, Duderhama, Ganderbal, J&K, India. Email:

ORCID ID:

**File I: Details of Analyses**

**Skewness and Kurtosis**

|  | |
| --- | --- |
|  | **THLE** |
| N | 382 |
| Missing | 0 |
| Mean | 21.0 |
| Standard deviation | 13.7 |
| Skewness | 0.392 |
| Std. error skewness | 0.125 |
| Kurtosis | -0.654 |
| Std. error kurtosis | 0.249 |

^Z^skewness = 3.14

^Z^kurtosis = −2.63

**Confirmatory Factor Analysis of the Four-Factor Structure of the Launay-Slade Hallucination Scale-Extended (N = 382)**

| **Factor** | **Item** | **Standardized Loading (λ)** | **SE** | **z** | **p** |
| --- | --- | --- | --- | --- | --- |
| Intrusive Thoughts | Q1 | .629 | — | — | — |
|  | Q2 | .805 | 0.126 | 10.30 | < .001 |
|  | Q3 | .637 | 0.113 | 9.55 | < .001 |
| Vivid Daydreams | Q5 | .718 | — | — | — |
|  | Q6 | .791 | 0.085 | 13.69 | < .001 |
|  | Q7 | .741 | 0.079 | 12.99 | < .001 |
| Multisensory HLE | Q11 | .695 | — | — | — |
|  | Q12 | .637 | 0.088 | 11.40 | < .001 |
|  | Q13 | .536 | 0.082 | 9.58 | < .001 |
|  | Q14 | .614 | 0.076 | 10.88 | < .001 |
|  | Q15 | .745 | 0.073 | 13.11 | < .001 |
| Auditory–Visual HLE | Q4 | .624 | — | — | — |
|  | Q8 | .651 | 0.097 | 10.69 | < .001 |
|  | Q9 | .637 | 0.091 | 10.42 | < .001 |
|  | Q10 | .701 | 0.083 | 11.24 | < .001 |
|  | Q16 | .718 | 0.090 | 11.33 | < .001 |

**Model Fit Indices**

| **Model** | **χ²** | **df** | **CFI** | **TLI** | **SRMR** | **RMSEA** | **90% CI for RMSEA** |
| --- | --- | --- | --- | --- | --- | --- | --- |
| Correlated four-factor model | 273 | 98 | .916 | .898 | .042 | .070 | [.061, .080] |
| Hierarchical four-factor model | 294 | 100 | .912 | .894 | .044 | .071 | [.062, .081] |

**Inter-Factor Correlations**

| **Factor** | **1** | **2** | **3** | **4** |
| --- | --- | --- | --- | --- |
| 1. Intrusive Thoughts | — |  |  |  |
| 2. Vivid Daydreams | .646*** | — |  |  |
| 3. Multisensory HLE | .580*** | .793*** | — |  |
| 4. Auditory–Visual HLE | .615*** | .841*** | .969*** | — |

**Reliability for factor structure**

|  | **Cronbach's α** |
| --- | --- |
| Intrusive Thoughts | 0.73 |
| Vivid Day Dreams | 0.79 |
| Multisensory HLE | 0.78 |
| Auditory-Visual HLE | 0.79 |
| Scale | 0.90 |

**Latent Class Prevalence Estimates**

| **Latent Class** | **Probability** | **Percentage** |
| --- | --- | --- |
| Class 1 (High Endorsement) | .213 | 21.3% |
| Class 2 (Moderate Endorsement) | .311 | 31.1% |
| Class 3 (Low Endorsement) | .476 | 47.6% |

**Latent Class Analysis: 2-Class Model**

| **Model fit** | | | | | | | |
| --- | --- | --- | --- | --- | --- | --- | --- |
| **Class** | **Log-likelihood** | **AIC** | **CAIC** | **BIC** | **Entropy** | **df** | **G²** |
| 2 | -7610 | 15478 | 16116 | 15987 | 0.938 | 252 | 10842 |

| **Marginal prevalences for Latent Class** | |
| --- | --- |
|  | **Probability** |
| **Class 1** | 0.475 |
| **Class 2** | 0.525 |

## Item response probabilities

| **Probability of Q1** | | | | | |
| --- | --- | --- | --- | --- | --- |
|  | **Y = 1** | **Y = 2** | **Y = 3** | **Y = 4** | **Y = 5** |
| Class 1 | 0.0713 | 0.110 | 0.218 | 0.437 | 0.1636 |
| Class 2 | 0.3646 | 0.195 | 0.107 | 0.273 | 0.0614 |

| **Probability of Q2** | | | | | |
| --- | --- | --- | --- | --- | --- |
|  | **Y = 1** | **Y = 2** | **Y = 3** | **Y = 4** | **Y = 5** |
| Class 1 | 0.0330 | 0.0834 | 0.194 | 0.435 | 0.2547 |
| Class 2 | 0.3444 | 0.1839 | 0.133 | 0.250 | 0.0885 |

| **Probability of Q3** | | | | | |
| --- | --- | --- | --- | --- | --- |
|  | **Y = 1** | **Y = 2** | **Y = 3** | **Y = 4** | **Y = 5** |
| Class 1 | 0.0504 | 0.130 | 0.1217 | 0.387 | 0.311 |
| Class 2 | 0.3136 | 0.187 | 0.0793 | 0.273 | 0.147 |

| **Probability of Q4** | | | | | |
| --- | --- | --- | --- | --- | --- |
|  | **Y = 1** | **Y = 2** | **Y = 3** | **Y = 4** | **Y = 5** |
| Class 1 | 0.156 | 0.249 | 0.1950 | 0.2296 | 0.17024 |
| Class 2 | 0.767 | 0.103 | 0.0578 | 0.0663 | 0.00540 |

| **Probability of Q5** | | | | | |
| --- | --- | --- | --- | --- | --- |
|  | **Y = 1** | **Y = 2** | **Y = 3** | **Y = 4** | **Y = 5** |
| Class 1 | 0.107 | 0.268 | 0.2645 | 0.2877 | 0.0728 |
| Class 2 | 0.711 | 0.122 | 0.0896 | 0.0486 | 0.0288 |

| **Probability of Q6** | | | | | |
| --- | --- | --- | --- | --- | --- |
|  | **Y = 1** | **Y = 2** | **Y = 3** | **Y = 4** | **Y = 5** |
| Class 1 | 0.111 | 0.264 | 0.1839 | 0.2976 | 0.14354 |
| Class 2 | 0.748 | 0.125 | 0.0729 | 0.0447 | 0.00964 |

| **Probability of Q7** | | | | | |
| --- | --- | --- | --- | --- | --- |
|  | **Y = 1** | **Y = 2** | **Y = 3** | **Y = 4** | **Y = 5** |
| Class 1 | 0.112 | 0.259 | 0.3121 | 0.2093 | 0.1082 |
| Class 2 | 0.722 | 0.150 | 0.0664 | 0.0448 | 0.0167 |

| **Probability of Q8** | | | | | |
| --- | --- | --- | --- | --- | --- |
|  | **Y = 1** | **Y = 2** | **Y = 3** | **Y = 4** | **Y = 5** |
| Class 1 | 0.143 | 0.235 | 0.2236 | 0.2739 | 0.1245 |
| Class 2 | 0.733 | 0.147 | 0.0369 | 0.0462 | 0.0369 |

| **Probability of Q9** | | | | | |
| --- | --- | --- | --- | --- | --- |
|  | **Y = 1** | **Y = 2** | **Y = 3** | **Y = 4** | **Y = 5** |
| Class 1 | 0.205 | 0.2761 | 0.1853 | 0.2397 | 0.0940 |
| Class 2 | 0.822 | 0.0841 | 0.0467 | 0.0323 | 0.0146 |

| **Probability of Q10** | | | | | |
| --- | --- | --- | --- | --- | --- |
|  | **Y = 1** | **Y = 2** | **Y = 3** | **Y = 4** | **Y = 5** |
| Class 1 | 0.272 | 0.3200 | 0.1872 | 0.13726 | 0.08311 |
| Class 2 | 0.916 | 0.0494 | 0.0250 | 0.00535 | 0.00452 |

| **Probability of Q11** | | | | | |
| --- | --- | --- | --- | --- | --- |
|  | **Y = 1** | **Y = 2** | **Y = 3** | **Y = 4** | **Y = 5** |
| Class 1 | 0.160 | 0.258 | 0.2105 | 0.2276 | 0.1436 |
| Class 2 | 0.748 | 0.115 | 0.0687 | 0.0482 | 0.0196 |

| **Probability of Q12** | | | | | |
| --- | --- | --- | --- | --- | --- |
|  | **Y = 1** | **Y = 2** | **Y = 3** | **Y = 4** | **Y = 5** |
| Class 1 | 0.113 | 0.136 | 0.2183 | 0.340 | 0.1934 |
| Class 2 | 0.622 | 0.126 | 0.0866 | 0.111 | 0.0543 |

| **Probability of Q13** | | | | | |
| --- | --- | --- | --- | --- | --- |
|  | **Y = 1** | **Y = 2** | **Y = 3** | **Y = 4** | **Y = 5** |
| Class 1 | 0.171 | 0.2290 | 0.1996 | 0.2828 | 0.1172 |
| Class 2 | 0.753 | 0.0819 | 0.0587 | 0.0780 | 0.0285 |

| **Probability of Q14** | | | | | |
| --- | --- | --- | --- | --- | --- |
|  | **Y = 1** | **Y = 2** | **Y = 3** | **Y = 4** | **Y = 5** |
| Class 1 | 0.196 | 0.276 | 0.2488 | 0.1796 | 0.09913 |
| Class 2 | 0.736 | 0.134 | 0.0540 | 0.0668 | 0.00996 |

| **Probability of Q15** | | | | | |
| --- | --- | --- | --- | --- | --- |
|  | **Y = 1** | **Y = 2** | **Y = 3** | **Y = 4** | **Y = 5** |
| Class 1 | 0.207 | 0.2905 | 0.2301 | 0.1997 | 0.0724 |
| Class 2 | 0.870 | 0.0810 | 0.0110 | 0.0236 | 0.0142 |

| **Probability of Q16** | | | | | |
| --- | --- | --- | --- | --- | --- |
|  | **Y = 1** | **Y = 2** | **Y = 3** | **Y = 4** | **Y = 5** |
| Class 1 | 0.183 | 0.3097 | 0.1917 | 0.2383 | 0.0771 |
| Class 2 | 0.897 | 0.0337 | 0.0458 | 0.0236 | 1.26e-73 |

## Profile plot

# Latent Class Analysis: 3-Class Model

| **Model fit** | | | | | | | |
| --- | --- | --- | --- | --- | --- | --- | --- |
| **Class** | **Log-likelihood** | **AIC** | **CAIC** | **BIC** | **Entropy** | **df** | **G²** |
| 3 | -7359 | 15107 | 16066 | 15872 | 0.952 | 187 | 10341 |

| **Marginal prevalences for Latent Class** | |
| --- | --- |
|  | **Probability** |
| **Class 1** | 0.216 |
| **Class 2** | 0.310 |
| **Class 3** | 0.474 |

## Item response probabilities

| **Probability of Q1** | | | | | |
| --- | --- | --- | --- | --- | --- |
|  | **Y = 1** | **Y = 2** | **Y = 3** | **Y = 4** | **Y = 5** |
| Class 1 | 0.0906 | 2.84e-18 | 0.110 | 0.535 | 0.2643 |
| Class 2 | 0.0797 | 0.199 | 0.269 | 0.351 | 0.1015 |
| Class 3 | 0.3817 | 0.196 | 0.111 | 0.267 | 0.0451 |

| **Probability of Q2** | | | | | |
| --- | --- | --- | --- | --- | --- |
|  | **Y = 1** | **Y = 2** | **Y = 3** | **Y = 4** | **Y = 5** |
| Class 1 | 0.0460 | 4.44e-25 | 0.0376 | 0.515 | 0.4011 |
| Class 2 | 0.0275 | 0.145 | 0.3011 | 0.362 | 0.1640 |
| Class 3 | 0.3755 | 0.192 | 0.1282 | 0.241 | 0.0634 |

| **Probability of Q3** | | | | | |
| --- | --- | --- | --- | --- | --- |
|  | **Y = 1** | **Y = 2** | **Y = 3** | **Y = 4** | **Y = 5** |
| Class 1 | 0.0367 | 0.0359 | 0.0728 | 0.343 | 0.512 |
| Class 2 | 0.0619 | 0.1945 | 0.1504 | 0.401 | 0.192 |
| Class 3 | 0.3406 | 0.1933 | 0.0783 | 0.272 | 0.116 |

| **Probability of Q4** | | | | | |
| --- | --- | --- | --- | --- | --- |
|  | **Y = 1** | **Y = 2** | **Y = 3** | **Y = 4** | **Y = 5** |
| Class 1 | 0.251 | 0.119 | 0.0988 | 0.3177 | 0.21361 |
| Class 2 | 0.163 | 0.321 | 0.2477 | 0.1555 | 0.11281 |
| Class 3 | 0.785 | 0.100 | 0.0524 | 0.0573 | 0.00556 |

| **Probability of Q5** | | | | | |
| --- | --- | --- | --- | --- | --- |
|  | **Y = 1** | **Y = 2** | **Y = 3** | **Y = 4** | **Y = 5** |
| Class 1 | 0.108 | 0.163 | 0.1790 | 0.3696 | 0.18064 |
| Class 2 | 0.152 | 0.343 | 0.2979 | 0.1982 | 0.00853 |
| Class 3 | 0.746 | 0.104 | 0.0880 | 0.0443 | 0.01706 |

| **Probability of Q6** | | | | | |
| --- | --- | --- | --- | --- | --- |
|  | **Y = 1** | **Y = 2** | **Y = 3** | **Y = 4** | **Y = 5** |
| Class 1 | 0.132 | 0.0566 | 0.0893 | 0.4609 | 0.2609 |
| Class 2 | 0.137 | 0.4023 | 0.2383 | 0.1681 | 0.0547 |
| Class 3 | 0.789 | 0.1141 | 0.0685 | 0.0279 | 1.80e-27 |

| **Probability of Q7** | | | | | |
| --- | --- | --- | --- | --- | --- |
|  | **Y = 1** | **Y = 2** | **Y = 3** | **Y = 4** | **Y = 5** |
| Class 1 | 0.187 | 0.0690 | 0.1641 | 0.3273 | 0.25269 |
| Class 2 | 0.111 | 0.3843 | 0.3845 | 0.1109 | 0.00885 |
| Class 3 | 0.754 | 0.1423 | 0.0602 | 0.0378 | 0.00611 |

| **Probability of Q8** | | | | | |
| --- | --- | --- | --- | --- | --- |
|  | **Y = 1** | **Y = 2** | **Y = 3** | **Y = 4** | **Y = 5** |
| Class 1 | 0.149 | 0.159 | 0.0823 | 0.3817 | 0.2284 |
| Class 2 | 0.184 | 0.267 | 0.2961 | 0.1779 | 0.0751 |
| Class 3 | 0.768 | 0.151 | 0.0338 | 0.0355 | 0.0124 |

| **Probability of Q9** | | | | | |
| --- | --- | --- | --- | --- | --- |
|  | **Y = 1** | **Y = 2** | **Y = 3** | **Y = 4** | **Y = 5** |
| Class 1 | 0.207 | 0.1339 | 0.1761 | 0.3376 | 0.14584 |
| Class 2 | 0.265 | 0.3512 | 0.1888 | 0.1360 | 0.05879 |
| Class 3 | 0.848 | 0.0792 | 0.0337 | 0.0333 | 0.00553 |

| **Probability of Q10** | | | | | |
| --- | --- | --- | --- | --- | --- |
|  | **Y = 1** | **Y = 2** | **Y = 3** | **Y = 4** | **Y = 5** |
| Class 1 | 0.419 | 0.1505 | 0.0926 | 0.1810 | 0.1573 |
| Class 2 | 0.250 | 0.4082 | 0.2227 | 0.0934 | 0.0255 |
| Class 3 | 0.933 | 0.0398 | 0.0274 | 1.69e-17 | 6.84e-143 |

| **Probability of Q11** | | | | | |
| --- | --- | --- | --- | --- | --- |
|  | **Y = 1** | **Y = 2** | **Y = 3** | **Y = 4** | **Y = 5** |
| Class 1 | 0.248 | 0.0461 | 0.0431 | 0.3454 | 0.3172 |
| Class 2 | 0.159 | 0.3977 | 0.2992 | 0.1291 | 0.0154 |
| Class 3 | 0.772 | 0.1053 | 0.0717 | 0.0398 | 0.0111 |

| **Probability of Q12** | | | | | |
| --- | --- | --- | --- | --- | --- |
|  | **Y = 1** | **Y = 2** | **Y = 3** | **Y = 4** | **Y = 5** |
| Class 1 | 0.190 | 0.0503 | 0.0903 | 0.317 | 0.3517 |
| Class 2 | 0.109 | 0.1922 | 0.2985 |  | 0.0831 |
| Class 3 | 0.643 | 0.1274 | 0.0783 | 0.112 | 0.0394 |

| **Probability of Q13** | | | | | |
| --- | --- | --- | --- | --- | --- |
|  | **Y = 1** | **Y = 2** | **Y = 3** | **Y = 4** | **Y = 5** |
| Class 1 | 0.4085 | 0.0555 | 0.1036 | 0.2331 | 0.1994 |
| Class 2 | 0.0755 | 0.3386 | 0.2486 | 0.2855 | 0.0518 |
| Class 3 | 0.7700 | 0.0734 | 0.0552 | 0.0770 | 0.0243 |

| **Probability of Q14** | | | | | |
| --- | --- | --- | --- | --- | --- |
|  | **Y = 1** | **Y = 2** | **Y = 3** | **Y = 4** | **Y = 5** |
| Class 1 | 0.346 | 0.125 | 0.1377 | 0.2520 | 0.1399 |
| Class 2 | 0.153 | 0.387 | 0.2943 | 0.1104 | 0.0547 |
| Class 3 | 0.753 | 0.115 | 0.0539 | 0.0670 | 0.0109 |

| **Probability of Q15** | | | | | |
| --- | --- | --- | --- | --- | --- |
|  | **Y = 1** | **Y = 2** | **Y = 3** | **Y = 4** | **Y = 5** |
| Class 1 | 0.321 | 0.0723 | 0.1159 | 0.3086 | 0.18205 |
| Class 2 | 0.183 | 0.4278 | 0.2748 | 0.1062 | 0.00829 |
| Class 3 | 0.905 | 0.0681 | 0.0103 | 0.0164 | 2.10e-12 |

| **Probability of Q16** | | | | | |
| --- | --- | --- | --- | --- | --- |
|  | **Y = 1** | **Y = 2** | **Y = 3** | **Y = 4** | **Y = 5** |
| Class 1 | 0.386 | 0.0525 | 0.0986 | 0.3289 | 0.1341 |
| Class 2 | 0.129 | 0.4526 | 0.2447 | 0.1491 | 0.0248 |
| Class 3 | 0.917 | 0.0277 | 0.0379 | 0.0176 | 1.44e-95 |

Profile plot

# Latent Class Analysis: 4- Class Model

Instructions

## Model information

Call:

glca::glca(formula = formula, data = data, nclass = nc, seed = 1)

Manifest items : Q1 Q2 Q3 Q4 Q5 Q6 Q7 Q8 Q9 Q10 Q11 Q12 Q13 Q14 Q15 Q16

Model : Latent class analysis

Number of latent classes : 4

Number of observations : 382

Number of parameters : 259

log-likelihood : -7178

G-squared : 9979

AIC : 14874

BIC : 15896

| **Model fit** | | | | | | | |
| --- | --- | --- | --- | --- | --- | --- | --- |
| **Class** | **Log-likelihood** | **AIC** | **CAIC** | **BIC** | **Entropy** | **df** | **G²** |
| 4 | -7178 | 14874 | 16155 | 15896 | 0.940 | 122 | 9979 |

| **Absolute model fit** | | | | | | | |
| --- | --- | --- | --- | --- | --- | --- | --- |
| **Class** | **Log-likelihood** | **AIC** | **CAIC** | **BIC** | **Entropy** | **df** | **G²** |
| 2 | -7610 | 15478 | 16116 | 15987 | 0.938 | 252 | 10842 |
| 3 | -7359 | 15107 | 16066 | 15872 | 0.952 | 187 | 10341 |
| 4 | -7178 | 14874 | 16155 | 15896 | 0.940 | 122 | 9979 |

| **Marginal prevalences for Latent Class** | |
| --- | --- |
|  | **Probability** |
| **Class 1** | 0.261 |
| **Class 2** | 0.337 |
| **Class 3** | 0.244 |
| **Class 4** | 0.158 |

## Item response probabilities

| **Probability of Q1** | | | | | |
| --- | --- | --- | --- | --- | --- |
|  | **Y = 1** | **Y = 2** | **Y = 3** | **Y = 4** | **Y = 5** |
| Class 1 | 0.0350 | 0.106 | 0.2662 | 0.452 | 0.1407 |
| Class 2 | 0.4364 | 0.158 | 0.0788 | 0.264 | 0.0622 |
| Class 3 | 0.2183 | 0.302 | 0.1865 | 0.267 | 0.0267 |
| Class 4 | 0.0999 | 7.91e-26 | 0.1147 | 0.496 | 0.2891 |

| **Probability of Q2** | | | | | |
| --- | --- | --- | --- | --- | --- |
|  | **Y = 1** | **Y = 2** | **Y = 3** | **Y = 4** | **Y = 5** |
| Class 1 | 0.0123 | 0.0399 | 0.2254 | 0.476 | 0.2462 |
| Class 2 | 0.4201 | 0.1626 | 0.1169 | 0.215 | 0.0851 |
| Class 3 | 0.1737 | 0.2808 | 0.2191 | 0.326 | 3.31e-53 |
| Class 4 | 0.0589 | 0.0160 | 0.0671 | 0.387 | 0.4713 |

| **Probability of Q3** | | | | | |
| --- | --- | --- | --- | --- | --- |
|  | **Y = 1** | **Y = 2** | **Y = 3** | **Y = 4** | **Y = 5** |
| Class 1 | 0.0243 | 0.1302 | 0.1614 | 0.517 | 0.167 |
| Class 2 | 0.3945 | 0.1408 | 0.0672 | 0.255 | 0.143 |
| Class 3 | 0.1714 | 0.2986 | 0.1095 | 0.303 | 0.118 |
| Class 4 | 0.0474 | 0.0345 | 0.0504 | 0.205 | 0.662 |

| **Probability of Q4** | | | | | |
| --- | --- | --- | --- | --- | --- |
|  | **Y = 1** | **Y = 2** | **Y = 3** | **Y = 4** | **Y = 5** |
| Class 1 | 0.0757 | 0.2831 | 0.1965 | 0.2908 | 0.15378 |
| Class 2 | 0.8601 | 0.0401 | 0.0148 | 0.0781 | 0.00686 |
| Class 3 | 0.4501 | 0.2700 | 0.1965 | 0.0555 | 0.02789 |
| Class 4 | 0.3620 | 0.1233 | 0.1189 | 0.1778 | 0.21796 |

| **Probability of Q5** | | | | | |
| --- | --- | --- | --- | --- | --- |
|  | **Y = 1** | **Y = 2** | **Y = 3** | **Y = 4** | **Y = 5** |
| Class 1 | 0.0489 | 0.2276 | 0.3411 | 0.3724 | 0.0100 |
| Class 2 | 0.8188 | 0.0416 | 0.0844 | 0.0397 | 0.0155 |
| Class 3 | 0.4654 | 0.3680 | 0.1152 | 0.0515 | 1.54e-95 |
| Class 4 | 0.1398 | 0.1767 | 0.1717 | 0.2470 | 0.2648 |

| **Probability of Q6** | | | | | |
| --- | --- | --- | --- | --- | --- |
|  | **Y = 1** | **Y = 2** | **Y = 3** | **Y = 4** | **Y = 5** |
| Class 1 | 0.0776 | 0.2473 | 0.2392 | 0.3463 | 0.0897 |
| Class 2 | 0.8593 | 0.0557 | 0.0617 | 0.0233 | 1.06e-112 |
| Class 3 | 0.4501 | 0.3976 | 0.0966 | 0.0557 | 7.38e00-8 |
| Class 4 | 0.1621 | 0.0684 | 0.1190 | 0.3352 | 0.3153 |

| **Probability of Q7** | | | | | |
| --- | --- | --- | --- | --- | --- |
|  | **Y = 1** | **Y = 2** | **Y = 3** | **Y = 4** | **Y = 5** |
| Class 1 | 0.0549 | 0.2838 | 0.4040 | 0.2572 | 2.13e0-44 |
| Class 2 | 0.8550 | 0.0687 | 0.0375 | 0.0389 | 7.50e-110 |
| Class 3 | 0.3591 | 0.3736 | 0.1878 | 0.0580 | 0.0215 |
| Class 4 | 0.2661 | 0.0837 | 0.1219 | 0.1808 | 0.3475 |

| **Probability of Q8** | | | | | |
| --- | --- | --- | --- | --- | --- |
|  | **Y = 1** | **Y = 2** | **Y = 3** | **Y = 4** | **Y = 5** |
| Class 1 | 0.0882 | 0.2222 | 0.3193 | 0.3183 | 0.0521 |
| Class 2 | 0.8938 | 0.0481 | 0.0166 | 0.0414 | 3.88e-20 |
| Class 3 | 0.4117 | 0.3901 | 0.1273 | 0.0279 | 0.0430 |
| Class 4 | 0.1799 | 0.1211 | 0.0354 | 0.3194 | 0.3442 |

| **Probability of Q9** | | | | | |
| --- | --- | --- | --- | --- | --- |
|  | **Y = 1** | **Y = 2** | **Y = 3** | **Y = 4** | **Y = 5** |
| Class 1 | 0.168 | 0.3363 | 0.18508 | 0.2501 | 0.0609 |
| Class 2 | 0.936 | 0.0196 | 0.00805 | 0.0363 | 0.0000 |
| Class 3 | 0.593 | 0.2552 | 0.10531 | 0.0348 | 0.0113 |
| Class 4 | 0.159 | 0.1181 | 0.22648 | 0.2834 | 0.2130 |

| **Probability of Q10** | | | | | |
| --- | --- | --- | --- | --- | --- |
|  | **Y = 1** | **Y = 2** | **Y = 3** | **Y = 4** | **Y = 5** |
| Class 1 | 0.130 | 0.392 | 0.2567 | 0.1804 | 0.0405 |
| Class 2 | 1.000 | 2.27e-32 | 4.45e-57 | 1.13e-263 | 8.68e0-96 |
| Class 3 | 0.653 | 0.235 | 0.0777 | 0.0338 | 3.87e-154 |
| Class 4 | 0.505 | 0.115 | 0.1016 | 0.0802 | 0.1979 |

| **Probability of Q11** | | | | | |
| --- | --- | --- | --- | --- | --- |
|  | **Y = 1** | **Y = 2** | **Y = 3** | **Y = 4** | **Y = 5** |
| Class 1 | 0.0449 | 0.2869 | 0.3068 | 0.2933 | 0.0681 |
| Class 2 | 0.8335 | 0.0429 | 0.0526 | 0.0554 | 0.0155 |
| Class 3 | 0.4889 | 0.3360 | 0.1431 | 0.0320 | 7.28e-230 |
| Class 4 | 0.3602 | 0.0753 | 0.0214 | 0.1922 | 0.3509 |

| **Probability of Q12** | | | | | |
| --- | --- | --- | --- | --- | --- |
|  | **Y = 1** | **Y = 2** | **Y = 3** | **Y = 4** | **Y = 5** |
| Class 1 | 0.0297 | 0.1521 | 0.2386 | 0.466 | 0.1132 |
| Class 2 | 0.7320 | 0.0740 | 0.0473 | 0.101 | 0.0456 |
| Class 3 | 0.3440 | 0.2391 | 0.2369 | 0.168 | 0.0120 |
| Class 4 | 0.2618 | 0.0503 | 0.0834 | 0.146 | 0.4588 |

| **Probability of Q13** | | | | | |
| --- | --- | --- | --- | --- | --- |
|  | **Y = 1** | **Y = 2** | **Y = 3** | **Y = 4** | **Y = 5** |
| Class 1 | 0.0221 | 0.2293 | 0.25979 | 0.4187 | 0.0700 |
| Class 2 | 0.8588 | 0.0369 | 0.00819 | 0.0705 | 0.0255 |
| Class 3 | 0.3994 | 0.2649 | 0.18174 | 0.1426 | 0.0114 |
| Class 4 | 0.5315 | 0.0944 | 0.06775 | 0.0471 | 0.2592 |

| **Probability of Q14** | | | | | |
| --- | --- | --- | --- | --- | --- |
|  | **Y = 1** | **Y = 2** | **Y = 3** | **Y = 4** | **Y = 5** |
| Class 1 | 0.103 | 0.2992 | 0.3077 | 0.2201 | 0.07013 |
| Class 2 | 0.863 | 0.0427 | 0.0239 | 0.0625 | 0.00776 |
| Class 3 | 0.371 | 0.3646 | 0.1774 | 0.0653 | 0.02182 |
| Class 4 | 0.449 | 0.1275 | 0.0942 | 0.1641 | 0.16500 |

| **Probability of Q15** | | | | | |
| --- | --- | --- | --- | --- | --- |
|  | **Y = 1** | **Y = 2** | **Y = 3** | **Y = 4** | **Y = 5** |
| Class 1 | 0.133 | 0.32513 | 0.262 | 0.2286 | 0.05089 |
| Class 2 | 0.987 | 0.00602 | 7.76e-111 | 1.24e-34 | 0.00684 |
| Class 3 | 0.486 | 0.33315 | 0.115 | 0.0656 | 0.00000 |
| Class 4 | 0.438 | 0.07870 | 0.118 | 0.1997 | 0.16616 |

| **Probability of Q16** | | | | | |
| --- | --- | --- | --- | --- | --- |
|  | **Y = 1** | **Y = 2** | **Y = 3** | **Y = 4** | **Y = 5** |
| Class 1 | 0.0391 | 0.33836 | 0.2269 | 0.32504 | 0.0706 |
| Class 2 | 0.9821 | 0.00845 | 3.29e-121 | 0.00943 | 0.0000 |
| Class 3 | 0.5429 | 0.24811 | 0.1656 | 0.04344 | 1.57e-111 |
| Class 4 | 0.5329 | 0.08340 | 0.0982 | 0.17041 | 0.1151 |

**File II: Informed Consent Form, Demographics and LSHS-E Questionnaire**

**Informed Participation Consent Form**

This study is being conducted for academic purposes to examine hallucination-like experiences among the general population. Participation involves completing a survey. There are no anticipated risks except possible mild discomfort. You may withdraw at any time. There is no monetary compensation for participation. All information collected will remain confidential and will be used strictly for academic and research purposes. The findings may be reported in chapters or research publications without revealing your identity. By participating in this study, you confirm that you are at least 18 years old and a resident of India. By selecting “I Agree”, you confirm that you have read and understood the information above and voluntarily consent to participate in this study.

**Demographics and Questionnaire**

| Age: | Gender: Male/Female | Educational Qualification: |
| --- | --- | --- |
| Occupation: Working/Not Working | Marital Status: Married/Single | Family Type: Nuclear/Joint |
| Domicile: Rural/Urban | Socioeconomic Status (Please answer as per Govt. Norms): APL/BPL | Religion: Hinduism/Islam/Christianity/Sikhism/Buddhism/Jainism |
| Having any diagnosed neurological, substance use or psychiatric disorder? | State or UT: |  |

**Instructions**

This survey is being conducted for research purposes to understand everyday perceptual experiences. Some individuals occasionally report unusual sensory experiences, even in the absence of an external stimulus. This questionnaire aims to assess such experiences in non-clinical populations. Please read each statement carefully and indicate how much it applies to you based on your typical experiences. There are no right or wrong answers. We are interested in your honest responses.

Use the following response scale:

0 – Certainly does not apply to me

1 – Possibly does not apply to me

2 – Unsure

3 – Possibly applies to me

4 – Certainly applies to me

| **S. No.** | **Statement** | **0**  Certainly does not apply to me | **1**  Possibly does not apply to me | **2**  Unsure | **3**  Possibly applies to me | **4**  Certainly applies to me |
| --- | --- | --- | --- | --- | --- | --- |
| 1. | Sometimes a passing thought will seem so real that it frightens me. | 0 | 1 | 2 | 3 | 4 |
| 2. | Sometimes my thoughts seem as real as actual events in my life. | 0 | 1 | 2 | 3 | 4 |
| 3. | No matter how hard I try to concentrate on my work, unrelated thoughts always creep into my mind. | 0 | 1 | 2 | 3 | 4 |
| 4. | In the past, I have had the experience of hearing a person's voice and then found that there was no-one there. | 0 | 1 | 2 | 3 | 4 |
| 5. | The sounds I hear in my daydreams are generally clear and distinct. | 0 | 1 | 2 | 3 | 4 |
| 6. | The people in my daydreams seem so true to life that I sometimes think that they are there. | 0 | 1 | 2 | 3 | 4 |
| 7. | In my daydreams I can hear the sound of a tune almost as clearly as if I were actually listening to it. | 0 | 1 | 2 | 3 | 4 |
| 8. | I often hear a voice speaking my thoughts aloud. | 0 | 1 | 2 | 3 | 4 |
| 9. | I have been troubled by hearing voices in my head. | 0 | 1 | 2 | 3 | 4 |
| 10. | On occasions I have seen a person's face in front of me when no-one was in fact there. | 0 | 1 | 2 | 3 | 4 |
| 11. | Sometimes, immediately prior to falling asleep or upon awakening, I have had the experience of having seen, felt or heard something or someone that wasn't there. | 0 | 1 | 2 | 3 | 4 |
| 12. | Sometimes, immediately prior to falling asleep or upon awakening, I have felt that I was floating or falling. | 0 | 1 | 2 | 3 | 4 |
| 13. | On certain occasions I have felt the presence of someone close who had passed away. | 0 | 1 | 2 | 3 | 4 |
| 14. | In the past, I have smelt a particular odour even though there was nothing there. | 0 | 1 | 2 | 3 | 4 |
| 15. | I have had the feeling of touching something or being touched and then found that nothing or no one was there. | 0 | 1 | 2 | 3 | 4 |
| 16. | Sometimes, I have seen objects or animals even though there was nothing there. | 0 | 1 | 2 | 3 | 4 |

If you feel uncomfortable after completing this survey, please contact 14416 or 1800-891-4416 for help.
